# Appendiceal Diverticulitis Presenting as Acute Appendicitis and Diagnosed After Appendectomy

**DOI:** 10.7759/cureus.23050

**Published:** 2022-03-10

**Authors:** Muhammer Ergenç, Tevfik Kıvılcım Uprak

**Affiliations:** 1 General Surgery, Istanbul Sultanbeyli State Hospital, Istanbul, TUR; 2 General Surgery, Marmara University School of Medicine, Istanbul, TUR

**Keywords:** diverticulosis of the appendix, appendiceal diverticulitis, diverticulitis of the appendix, diverticulitis, appendiceal diverticulosis, appendectomy, acute appendicitis, appendicular diverticulitis, diverticular disease of the appendix

## Abstract

Introduction

Diverticular disease of the appendix (DDA) is a rare appendiceal pathology. It is usually present similar to acute appendicitis. Because of its rarity, the DDA is poorly comprehended. This study evaluates the incidence, clinical and pathological characteristics of appendiceal diverticulitis diagnosed after appendectomy.

Methods

We performed a retrospective analysis of patients who underwent appendectomy between January 2016 and January 2022 at the Istanbul Sultanbeyli State Hospital General Surgery Clinic. The following parameters were analyzed: age and gender, preoperative diagnosis, laboratory results, radiological imaging findings, surgical technique, histopathological examination of specimens, and complications.

Results

A total of 1586 patients were analyzed. In the pathology, diverticular disease of the appendix was detected in 10 patients (0.63%). The DDA patients’ mean age was 34.4 years, and the male to female ratio was 4:1. We detected low-grade appendiceal mucinous neoplasia in one of our patients.

Conclusion

Appendiceal diverticulitis is rare and usually presents as acute appendicitis. Most DDAs are detected incidentally during the postoperative period and are associated with an increased risk of appendiceal neoplasm. Appendectomy specimens should be carefully examined histopathologically to detect diverticular disease of the appendix.

## Introduction

Inflammation of the vermiform appendix is ​​defined as appendicitis. Worldwide, acute appendicitis is one of the most common general surgical emergencies, so appendectomy is one of the most frequently performed surgical operations. Luminal obstruction of the appendix (by a fecalith, impacted stool, lymphoid hyperplasia; infrequently by a tumor) causes acute appendicitis, but not all reasons are known [1,2].

Diverticular disease of the appendix (DDA) is a rare appendiceal pathology and incidence of 0.004-2.1% in appendectomy specimens. DDA present similar to acute appendicitis. Classification of diverticula according to their origin is congenital or acquired, and classified based on histological criteria are true or false (or pseudo-diverticula), respectively. The best method for diagnosis is histopathology, so usually diagnosed after surgery [3-5]. Due to its rarity, the DDA is poorly comprehended, and there is insufficient literature data. This study aimed to evaluate the incidence, clinical and pathological characteristics of appendiceal diverticulitis diagnosed after appendectomy in rural areas.

## Materials and methods

Study design

We performed a retrospective evaluation of patients who underwent appendectomy between January 2016 and January 2022 at the secondary-care hospital general surgery clinic (Istanbul Sultanbeyli State Hospital). This study was approved by the Clinical Research Ethics Committee of Istanbul Kartal Dr. Lutfi Kırdar City Hospital (approval number: 2022/514/219/7).

Inclusion and exclusion criteria

Patients diagnosed with appendiceal diverticulitis through histopathological examination after appendectomy for suspected acute appendicitis were included in the study. Exclusion criteria were (1) patients with missing data, (2) appendectomies associated with other procedures, and (3) cases where the appendiceal diverticulum was not confirmed on pathology.

Data collection 

We used patients’ files and hospital records for data acquisition. The following parameters were analyzed: age and gender, preoperative diagnosis, laboratory results, radiological imaging findings, surgical technique, histopathological examination of specimens, and complications. Diverticula were classified according to the morphological types defined by Lipton et al.: type 1 - appendiceal diverticulitis and normal appendix; type 2 - appendiceal diverticulitis and acute appendicitis; type 3 - appendiceal diverticulum and acute appendicitis; type 4 - appendiceal diverticulum and normal appendix [6]. Types 1-3 were divided into subgroups with or without perforation.

Statistical analysis

We performed a statistical analysis using the Statistical Package for the Social Sciences (SPSS) version 24 for Mac (Armonk, NY: IBM Corp.). The frequency procedure was applied to the categorical variables. Normally, distributed data were expressed as mean ± standard deviation, and data not following the normal distribution were described as the median. The primary outcome of this study was to determine the incidence and clinicopathological features of appendiceal diverticulitis diagnosed after appendectomy in a secondary care hospital.

## Results

**Figure 1:**
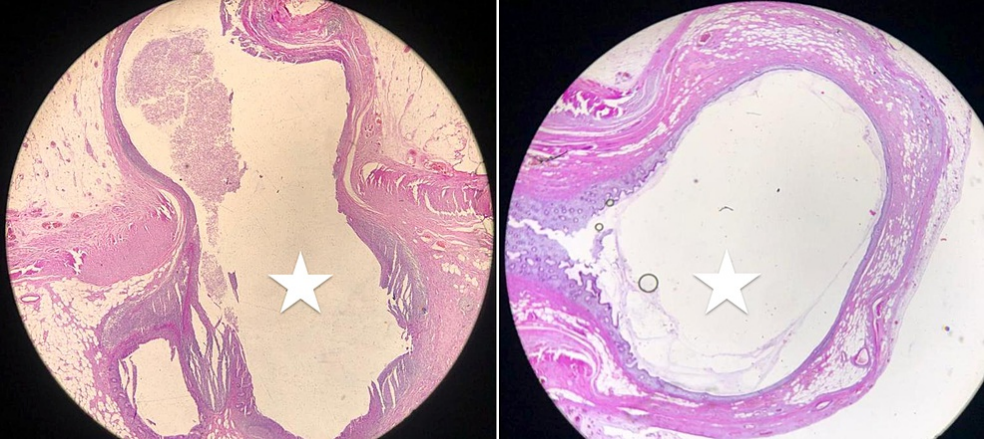
Histopathological images, sagittal sections, showing appendiceal diverticulitis at the tip of the appendix (white stars: diverticular lumen). Hematoxylin and eosin stain (H&E), 20x magnification.

All appendectomy procedures were evaluated. After three patients with missing detailed pathology reports were excluded, 1586 patients were analyzed. In the pathology, diverticular disease of the appendix was detected in 10 patients (0.63%) (Figure 1). The DDA patients’ mean age was 34.4 years, and the male to female ratio was 4:1 (Table 1).

**Table 1:** Demographics of patients diagnosed with appendiceal diverticulitis.

Parameters		Appendiceal diverticulitis n = 10
Age (years)	Mean (std. deviation)	34.4 (17.2)
	Minimum-maximum	15-63
Gender	Male	8 (80%)
	Female	2 (20%)

**Figure 2:**
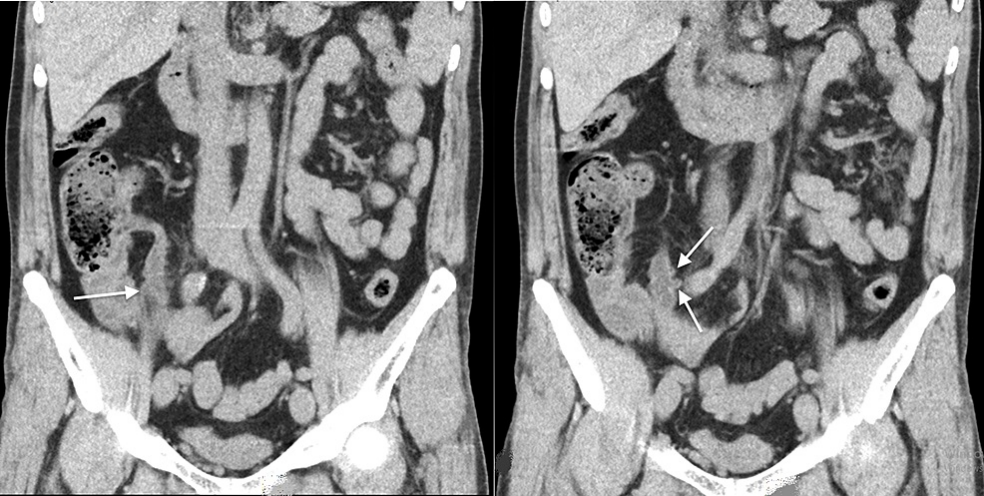
Coronal images of the abdominal computed tomography scan showing multiple diverticula of the appendix (white arrows).

All patients who presented with acute abdominal pain were evaluated. Computed tomography was utilized to examine nine patients, whereas ultrasonography was used in only one patient (Figure 2). Nine patients were operated on with the diagnosis of acute appendicitis and one patient with intra-abdominal perforation. Leukocytosis in seven patients and neutrophilia in five patients were observed.

The mean white blood count (WBC) of 13.5 x 10^9^/L (±5.02) and mean neutrophil percentage of 70.6 (±7.82) were detected (Table 2). After pathological examination, patients were diagnosed with diverticular disease of the appendix. According to Lipton classification, nine of our patients were classified as type 2 and one (18-year-old male) with type 2 plus perforation. The mean number of the diverticulum was 2.20 (±1.75). One young patient, sessile serrated adenoma, was seen as associated neoplasia, and low-grade appendiceal mucinous neoplasm was detected in an elderly patient. The median hospital stay was two (1-12) days. Significant complications requiring hospital readmission and 30-day mortality were not observed in any patient. Detailed information on the patients diagnosed with appendiceal diverticulitis is given in Table 2. 

**Table 2:** Characteristics of patients diagnosed with appendiceal diverticulitis. CT: computed tomography; AA: acute appendicitis; US: abdominal ultrasound imaging; OA: open appendectomy; WBC: white blood count (4-11 x 10^9^/L); NE%: percentage of neutrophils (50-70%)

Patient number	Age (years) /gender	Preoperative imaging/diagnosis	WBC	NE%	Surgery	Hospital stay (day)	Number of diverticulum	Associated neoplasms
1	14/male	CT/AA	14.5	69.8	OA	3	1	Sessile serrated adenoma
2	17/male	CT/AA	10.8	66.9	OA	1	1	No neoplasm
3	18/male	CT/abdominal perforation	26.2	74.9	Appendectomy (midline laparotomy)	12	1	No neoplasm
4	19/male	CT/AA	12.4	70.7	OA	3	3	No neoplasm
5	35/male	US/AA	10.5	66.4	OA	2	1	No neoplasm
6	35/male	CT/AA	9.3	56.5	OA	2	6	No neoplasm
7	41/male	CT/AA	12.0	67.2	OA	1	4	No neoplasm
8	46/male	CT/AA	11.3	87.2	OA	3	3	No neoplasm
9	56/female	CT/AA	11.1	73.2	Laparoscopic appendectomy	2	1	No neoplasm
10	63/female	CT/AA	17.6	73.4	OA	2	1	Low-grade appendiceal mucinous neoplasm

## Discussion

DDA often presents a clinically similar to acute appendicitis and is usually treated with appendectomy. Therefore, appendiceal diverticulitis is generally diagnosed after the pathological examination. DDA may be associated with complicated appendicitis [5,7].

Male gender, older age, cystic fibrosis, and Hirschsprung's disease are known risk factors for DDA [3,5,8]. DDA is usually asymptomatic and often presents in the fourth and fifth decades. We found our DDA patients' mean age was 34.4 years; 40% of the patients were in the second decade of their life. The mean WBC of our patients was 13.5, but lower rates have been reported in the literature [3,7,9]. The incidence of appendiceal diverticulitis detected 0.63 in our study, consistent with national and international studies [3,4,10,11].

DDA is classified into two types: congenital and acquired. The acquired diverticulum is the most common type, and its incidence varies between 0.004% and 2.1%. The congenital diverticulum is very rare and reported with an incidence of 0.014% [8,12]. Acquired diverticula are often multiple and located in the distal 1/3 of the appendix at the mesenteric margin [10]. According to Lipton classification, type 2 was the most common in our study. However, there are studies in the literature in which types 1 and 3 were detected more frequently [3,5,7,13,14]. It has been reported that the rate of accompanying colonic diverticulum is high in patients with appendiceal diverticulum [7,15].

Since DDA and epithelial neoplasia have a relatively high correlation, a more detailed macroscopic and microscopic examination should be performed when the appendiceal diverticulum is detected [4,13]. We detected low-grade appendiceal mucinous neoplasia in one of our patients. Computed tomography is recommended compared to ultrasonography as the imaging method for diagnosis. The following parameters are significant for DDA: peri-appendiceal fat stranding, a larger appendiceal diameter, the saccular structure of the appendix wall, cecum or ascending colon diverticulum, and peri- appendiceal or peri-cecal fluid collection [11,15-18].

Elective appendectomy is recommended for incidentally detected DDA due to the high risk of diverticulitis, perforation, and development of appendiceal malignancy risk [7,8,11,15]. Our study has certain limitations. It is a single-center and low-volume study. The limited number of patients and retrospectively collected data resulted in a lack of some information.

## Conclusions

Appendiceal diverticulitis is rare and usually presents as acute appendicitis. Most DDAs are detected incidentally during the postoperative period and are associated with an increased risk of appendiceal neoplasm. Appendectomy specimens should be carefully examined histopathologically to detect diverticular disease of the appendix.
